# Peripheral administration of nanomicelle-encapsulated anti-Aβ oligomer fragment antibody reduces various toxic Aβ species in the brain

**DOI:** 10.1186/s12951-023-01772-y

**Published:** 2023-01-31

**Authors:** Akiko Amano, Nobuo Sanjo, Wataru Araki, Yasutaka Anraku, Makoto Nakakido, Etsuro Matsubara, Takami Tomiyama, Tetsuya Nagata, Kouhei Tsumoto, Kazunori Kataoka, Takanori Yokota

**Affiliations:** 1 Department of Neurology and Neurological Science, Graduate School of Medical and Dental Sciences, Tokyo Medical and Dental University, 1-5-45 Yushima Bunkyo-ku, Tokyo, 113-8510 Japan; 2 Department of Bioengineering, School of Engineering, The University of Tokyo, Tokyo, Japan; 3 Innovation Center of Nano Medicine, Kawasaki Institute of Industrial Promotion, Kanagawa, Japan; 4 Department of Chemistry and Biotechnology, School of Engineering, The University of Tokyo, Tokyo, Japan; 5 Department of Neurology, Oita University, Oita, Japan; 6 Department of Translational Neuroscience, Osaka Metropolitan University Graduate School of Medicine, Osaka, Japan; 7 The Institute of Medical Science, The University of Tokyo, Tokyo, Japan

**Keywords:** Alzheimer’s disease, Polymeric nanomicelle, Amyloid β oligomer, Pyroglutamated amyloid β

## Abstract

**Background:**

Although a large amount of evidence has revealed that amyloid β (Aβ), especially Aβ oligomers, protofibrils, and pyroglutamated Aβs, participate primarily in the pathophysiological processes of Alzheimer’s disease, most clinical trials of anti-Aβ antibody therapy have never acquired successful efficacy in human clinical trials, partly because peripheral administration of antibody medications was unable to deliver sufficient amounts of the molecules to the brain. Recently, we developed polymeric nanomicelles capable of passing through the blood–brain barrier that function as chaperones to deliver larger amounts of heavy molecules to the brain. Herein, we aimed to evaluate the efficacy of newly developed antibody 6H4 fragments specific to Aβ oligomers encapsulated in polymeric nanomicelles on the development of Alzheimer’s disease pathology in Alzheimer’s disease model mice at the age of emergence of early Alzheimer’s disease pathology.

**Results:**

During the 10-week administration of 6H4 antibody fragments in polymeric nanomicelles, a significant reduction in the amounts of various toxic Aβ species, such as Aβ oligomers, toxic Aβ conformers, and pyroglutamated Aβs in the brain was observed. In addition, immunohistochemistry indicated inhibition of diameters of Aβ plaques, Aβ-antibody immunoreactive areas, and also plaque core formation. Behavioral analysis of the mice model revealed that the 6H4 fragments-polymeric nanomicelle group was significantly better at maintaining long-term spatial reference memory in the probe and platform tests of the water maze, thereby indicating inhibition of the pathophysiological process of Alzheimer’s disease.

**Conclusions:**

The results indicated that the strategy of reducing toxic Aβ species in early dementia owing to Alzheimer’s disease by providing sufficient antibodies in the brain may modify Alzheimer’s disease progression.

**Supplementary Information:**

The online version contains supplementary material available at 10.1186/s12951-023-01772-y.

## Background

Alzheimer’s disease (AD) is the most common neurodegenerative disease that causes dementia; it is characterized pathologically by extraneural deposition of amyloid β (Aβ) continually cleaved from an amyloid precursor protein by β- and γ-secretase activities in the central nervous system. Growing evidence has shown that synaptic dysfunction associated with extraneural Aβ oligomers (AβOs) in the brains of patients with AD is one of the main toxic agents in the early stages of AD pathology [1]. Recently, the following newly identified toxic Aβ species have been reported: intraneural AβO [2], which is inversely correlated with synaptic density [3]; toxic Aβ conformers [4], which have a turn structure at positions 22–23 associated with rapid oligomerization, neurotoxicity, and synaptotoxicity; pyroglutamated Aβ (N3pE Aβ) [5, 6], amino-terminally truncated and pyroglutamate-modified Aβ with exceptionally high amyloidogenicity, proteolytic resistance, neurotoxicity, and accelerating senile plaque formation with other Aβ species. These toxic Aβ species cooperatively participate in the pathophysiology of AD, causing a cognitive decline in collaboration with abnormally phosphorylated tau proteins [7]. However, anti-Aβ therapy trials, including anti-AβO therapies and aducanumab, have never achieved satisfactory efficacy in humans [8, 9]. Possible reasons for these unsatisfactory results include the following: (1) inability of antibodies to selectively act against disease-specific targets in the brain, (2) presence of multiple targets involving various forms of Aβ, 3) relatively late administration of antibodies, (4) side effects (such as amyloid-related imaging abnormalities [ARIA; ARIA-E: suggestive of vasogenic edema and sulcal effusions, ARIA-H: suggestive of hemosiderin deposits]), and (5) extremely low permeability of the blood–brain barrier (BBB) to the movement of full-body antibodies from the blood vessels into the brain.

It is well known that delivering high molecular weight compounds, including full-body antibodies, into the brain is difficult [10] because only small molecules less than approximately 500 Da can pass through the BBB [11]. To resolve the difficulty of trans-BBB migration, we recently developed a peripheral intravenous administration of glucosylated polymeric nanomicelles (PMs) via glucose transporters in vascular endothelial cells and were successful in demonstrating that PMs that are more than 56 times larger compared with conventional methods can pass through the BBB [12]. Moreover, this technology was adopted for the anti-Aβ antibody 3D6 (Bapineuzumab) fragment (Fab), and the anti-Aβ antibody fragments (Fabs) encapsulated in the PMs effectively inhibited Aβ deposition and reduced neurotoxicity in the brains of young AD model mice [13].

Next, the anti-AβO antibody 6H4, which is compatible with the previously reported monoclonal anti-AβO antibody 72D9 [14], was newly generated. The antibody has specificity for the structure of AβO conformation although not the amino-acid sequence of Aβ. The antibody recognizes pentamers, trimers, and high-molecular-weight oligomeric polymers of Aβ. Interestingly, the Fabs maintained the same sensitivity and specificity to the AβO structure. To evaluate the efficacy of 6H4 Fabs encapsulated in the PMs on AD pathology, we evaluated the quantitative and histochemical changes of these toxic Aβ species after long-term peripheral administration of anti-AβO 6H4 Fabs encapsulated in the PMs in AD model mice at the age of amyloid plaque deposition in the brain. We also aimed to determine the cognitive function of these mice using a behavioral analysis.

## Results

### Biochemical analysis revealed a significant reduction of the toxic Aβ species

**Fig. 1:**
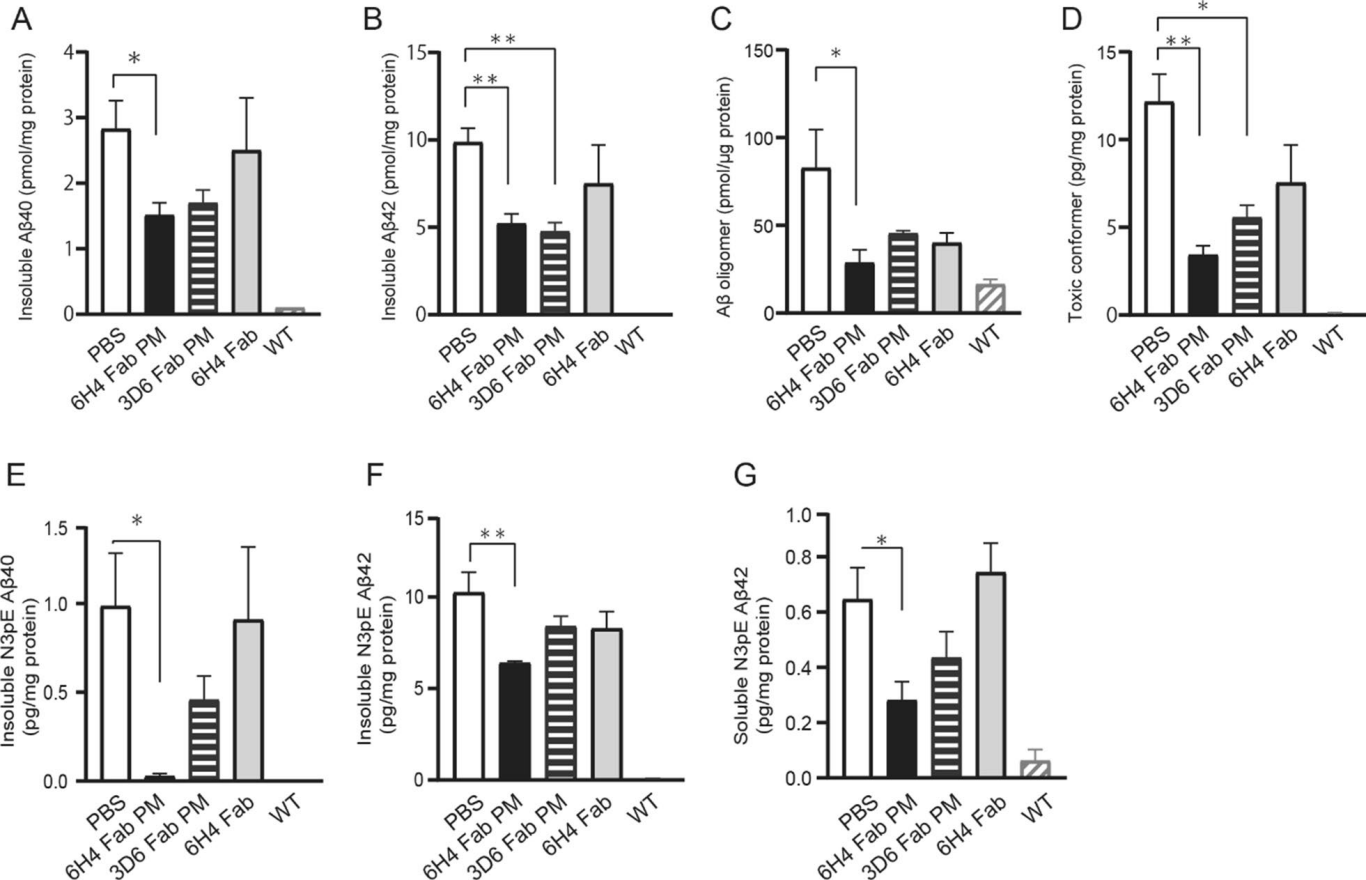
Quantitative analysis of Aβs and toxic Aβ species in mice brains. Quantitative evaluation of tris-buffered saline (TBS)-insoluble amyloid β (Aβ)40 and Aβ42 in brain homogenates of Alzheimer’s disease model mouse brains using enzyme-linked immunosorbent assay (ELISA) kits **A**, **B** revealed significant Aβ40 reduction in the 6H4 antibody fragment (Fab) polymeric nanomicelle (PM) group (black bar) compared with that in the phosphate-buffered saline (PBS) group (white bar) **A** and significant Aβ42 reduction in the 6H4 (black bar) and 3D6 Fab PM (horizontal striped bar) groups compared with that in the PBS group (white bar) (**B**). The quantitative dot-blot of Aβ oligomer (O) in mouse brain homogenates using 6H4 antibody revealed significant 6H4 Fab PM (black bar) reduction, and non-significant reduction in the 3D6 Fab PM (horizontal striped bar) and 6H4 Fab (gray bar) groups compared with the PBS group (white bar) (**C**). Toxic conformers in the mice brain homogenates measured using ELISA kits revealed significant reductions in the 6H4 (black bar) and 3D6 Fab PM (horizontal striped bar) groups compared with that in the PBS group (white bar) (**D**). N3pE Aβ species was measured in the brain homogenates of the 6H4 Fab PM group (white bars) using ELISA kits, revealing that the amounts of TBS-insoluble N3pE Aβ40s (**E**), TBS-insoluble N3pE Aβ42 (**F**), and TBS-soluble N3pE Aβ42 (**G**) were 0.06 ± 0.02, 6.4 ± 0.1, and 0.2 ± 0.06 pg/mg proteins, respectively, and were significantly lower than those of the PBS groups (white bars, 0.9 ± 0.3, 10.2 ± 1.0, and 0.6 ± 0.11, respectively). Values are expressed as the mean ± standard error of the mean. The hatched bar on the left indicates the wild-type (WT) group. Statistical evaluations were performed using one-way way analysis of variance with Tukey’s post-hoc test. *p < 0.05, **p < 0.01

In the 6H4 Fab group, the amounts of insoluble Aβ40 and Aβ42 in the mouse brain homogenates were measured using enzyme-linked immunosorbent assay (ELISA kits) were 47% (*p* < 0.05; Fig. 1A) and 47% (*p* < 0.01; Fig. 1B) lower than those in the phosphate-buffered saline (PBS) group, respectively. The amount of insoluble Aβ42 was significantly smaller in the 3D6 Fab PM group than that of the PBS group by 53% (Fig. 1B; *p* < 0.01). The amount of AβO in the 6H4 Fab PM group was 65% less than that in the PBS group (28.7 ± 7.3 vs. 82.7 ± 21.7 pmol/μg protein) (Fig. 1C; *p* < 0.05). Regarding toxic Aβ conformers, the amount of conformers in the insoluble fractions of the brain homogenates in the 6H4 Fab PM and 3D6 Fab PM groups was 72% (*p* < 0.01) and 54% (*p* < 0.05) less than that in the PBS group, respectively (Fig. 1D). The amounts of insoluble N3pE Aβ40s, insoluble N3pE Aβ42s, and soluble N3pE Aβ42s in the brain homogenates were significantly reduced only in the 6H4 Fab PM group when compared with the PBS group, by reductions of 93% (*p* < 0.05), 35% (*p* < 0.01), and 38% (*p* < 0.05), respectively (Fig. 1E–G). The amount of soluble N3pE Aβ40 in all groups was below the detection limit of the ELISA kit; the antibody may not have specifically recognized soluble N3pE Aβ40. The results clearly demonstrate that the peripheral administration of the 6H4 Fabs encapsulated in the PM could reduce the significant amounts of toxic Aβ species in the mouse brain without changing the total amounts of soluble Aβ40 and Aβ42 (see Additional file 1: Fig. S1A, B); this occurred by the 6H4 Fabs directly capturing the AβOs, including the toxic species (see Additional file 1: Fig. S1C). The amount of intravenously administered Fabs encapsulated in the PMs being delivered into the brain was evaluated using methods similar to those reported in our previous study [13] and confirmed in the present study: the rate of 6H4 and 3D6 Fabs reaching the brain was higher than that of 6H4 Fabs administered without PMs (see Additional file 1: Fig. S2).

### Immunohistochemistry revealed significant inhibition of Aβ deposition and Aβ core formation

**Fig. 2:**
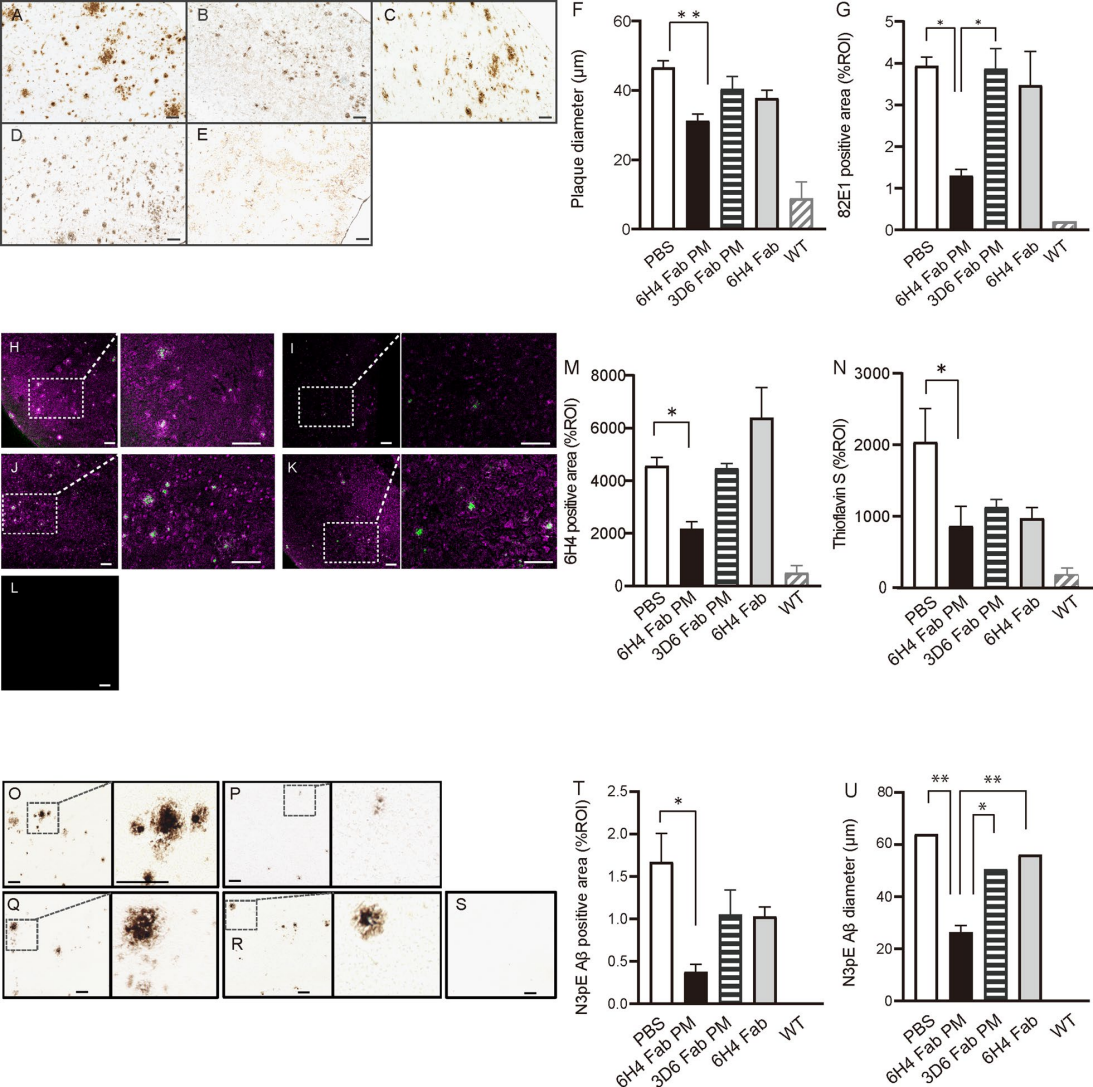
Quantitatively evaluation of immune-positivity of toxic Aβs in brain sections. After administering phosphate-buffered saline (PBS) in **A** anti-amyloid β oligomer (AβO) 6H4 antibody fragments (Fabs) (6H4 Fab PM) and **B** anti-Aβ 3D6 Fabs encapsulated in PMs (3D6 Fab PM), **C** anti-AβO 6H4 Fabs (6H4 Fab), and **D** Alzheimer’s disease (AD) model and **E** untreated wild-type (WT) mice, immunohistochemistry with anti-Aβ 82E1 antibody was performed. Panels A–E reveal significantly narrower plaque diameters **F** and smaller 82E1positive areas **G** in the 6H4 Fab PM group (black bars) than in the PBS group (white bars)**.** Images of immunofluorescent positive signals for anti-AβO 6H4 antibody (magenta) and thioflavin S (green) in AD mice treated with PBS (**H**), 6H4 Fab PMs (**I**), 3D6 Fab PM (**J**), and 6H4 Fab (**K**), and WT mice **(L).** Quantitative analysis of the % region of interest (ROI) for 6H4 antibody (**M**) and thioflavin S (**N**) positive signals of AD mice observed in panels H–L revealed significantly reduced %ROI and thioflavin S-positive signals in the 6H4 Fab PM group compared with that in the PBS group**.** Immunoreactive-images for anti-N3pE Aβ antibody in AD mice treated with PBS (**O**)**,** 6H4 Fab PM (**P**), 3D6 Fab PM (**Q**)**,** and 6H4 Fab (**R**) and WT mice (**S**). Enlarged views are presented inside dashed lines on each figure. N3pE Aβ-positive Aβ plaques in the 6H4 Fab PM group showed small diffuse-like plaques without Aβ cores (**P**). Quantitative values of the immunostained N3pE Aβs represent the average numbers of plaques in 10 figures in panels O–S. The 6H4 Fab PM group showed significantly smaller N3pE Aβ antibody-positive areas (**T**) and diameters (**U**) than those in the PBS group. Scale bar = 100 μm. One-way analysis of variance with Tukey’s post-hoc test was performed. Values = mean ± standard error of the mean. *p < 0.05, **p < 0.01

Immunohistochemical analysis demonstrated various amounts of anti-Aβ antibody (82E1)-immunoreactive plaques in mouse brain sections (Fig. 2A–G), and the diameters and total areas of anti-Aβ antibody-positive plaques were evaluated. The diameters of the plaques and the immunoreactive areas (% of region of interest [%ROI]) were significantly smaller and narrower in the 6H4 Fab PM group (Fig. 2B) than those in the PBS group (Fig. 2A) by 34% (*p* < 0.01; Fig. 2F) and 67% (*p* < 0.05; Fig. 2G), respectively. The comparative analysis of fluorescent immunopositivity of AβO in the mouse brain sections revealed that the fluorescent signals of AβOs in the 6H4 Fab PM group (Fig. 2I) were significantly fewer than those in the PBS group (Fig. 2H), which was confirmed statistically (*p* < 0.05; Fig. 2M). The fluorescent signals of AβOs in the 3D6 Fab PM (Fig. 2J) and 6H4 Fab without PM (Fig. 2K) groups were not significantly different from those in the PBS group (Fig. 2M). The immune-positive areas of thioflavin S, which is a commonly used aggregated protein marker which was previously reported to detect β-sheet rich structures [15], were significantly reduced only in the 6H4 Fab PM group (Fig. 2I), when compared with the PBS group (*p* < 0.05; Fig. 2H and N. The thioflavin S-positive areas in the 3D6 Fab PM (Fig. 2J) and 6H4 Fab groups (Fig. 2K) were also reduced, although not significantly (Fig. 2N). The total amount of AβO in the brain of the 3D6 Fab PM and 6H4 Fab groups (Fig. 1C and see Additional file 1: Fig. S3) was not necessarily compatible with the %ROIs of those groups (Fig. 2M); this was probably because dot-blotting measured soluble AβO, whereas immunohistochemistry measured both soluble and insoluble AβO in the frozen brain sections. The N3pE Aβ immunopositive areas in the PBS group identified Aβ plaques with dense cores (classic cores) [16], as shown in Fig. 2O. However, the N3pE Aβ immunopositive plaques in the brains of the 6H4 Fab PM group did not have dense cores (Fig. 2P). Similar to the PBS group, the 3D6 Fab PM (Fig. 2Q) and 6H4 Fab (Fig. 2R) groups also had dense plaque cores. These results indicated that the strong aggregative induction of N3pE Aβs was reduced qualitatively and quantitatively by anti-AβO 6H4 Fab administration in the brain. The reduction rates of the immunohistochemically positive N3pE Aβ areas per ROI and the N3pE Aβ antibody-positive plaque diameter in the 6H4 Fab PM group were 78% (*p* < 0.05; Fig. 2T) and 58% (*p* < 0.01; Fig. 2U), respectively, these were lower than the corresponding values in the PBS group.

### Behavioral analysis supported the efficacy of the anti-AβO Fabs encapsulated in the PMs

**Fig. 3:**
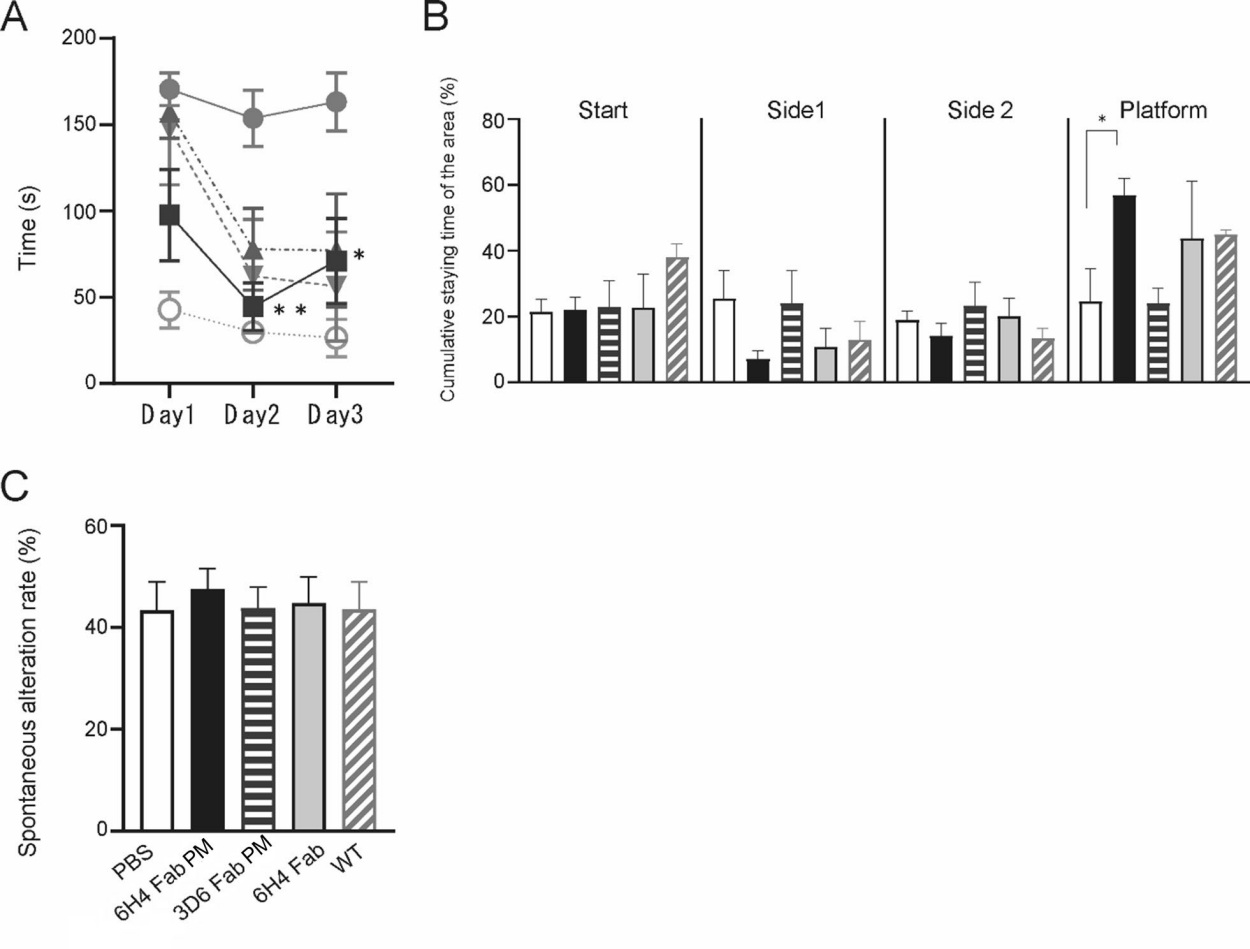
Comparative efficacies of the administration of various anti-Aβ Fabs.** A** The three-day hidden platform test of the AD model mice was performed after 10 weeks of PBS administration in anti-amyloid β oligomer (AβO) 6H4 Fabs encapsulated in the PMs, anti-Aβ 3D6 Fabs encapsulated in the PMs, and anti-AβO 6H4 Fabs. The 6H4 Fab PM group (black squares) required significantly shorter times to reach the platform compared with the PBS group (gray circles) on days 2 and 3. Those in the 3D6 Fab PM (black triangles) and 6H4 Fab (gray reverse triangles) groups showed no significant differences compared with the PBS group. **B** The 6H4 Fab PM group (black bars) stayed for the longest time in the platform quarter area in the water maze test, and the difference was significant. **C** No significant difference was observed among the groups in the spontaneous alternation rate in the Y-maze test. Values are expressed as the mean ± standard error of the mean. Two-way analysis of variance (ANOVA) with Dunnett's post hoc test was performed in **A** and one-way ANOVA with Dunnett's post hoc test in **B**, **C**. *p < 0.05, **p < 0.01. *Aβ* amyloid β, *Fab* antibody fragment, *PBS* phosphate-buffered saline, *PM* polymeric nanomicelle, *WT* wild type

To support the pathological and biochemical analyses, a few behavioral tests were performed. In the hidden platform test, the 6H4 Fab PM group demonstrated a significantly faster time to reach the hidden platform on day 2 (*p* < 0.01) and day 3 (*p* < 0.05) than the PBS group (Fig. 3A). It is suggested that long-term visuospatial learning ability of the mice was maintained owing to a reduction of the pathological deterioration observed in AD, by peripheral administration of the anti-AβO 6H4 Fabs encapsulated in the PMs. In the probe test, the cumulative time that mice stayed in the platform quadrant area compared with the other three quadrants was significantly longer in the 6H4 Fab PM group (57%) than that of the PBS group (24%) (*p* < 0.05; Fig. 3B). The spontaneous alternation rate of the Y-maze test, which indicates spatial reference memory, showed the highest value in the 6H4 Fab PM group, although the difference was not significant (Fig. 3C).

### Long-lasting Aβ aggregation inhibition by anti-Aβ oligomer antibody

**Fig. 4:**
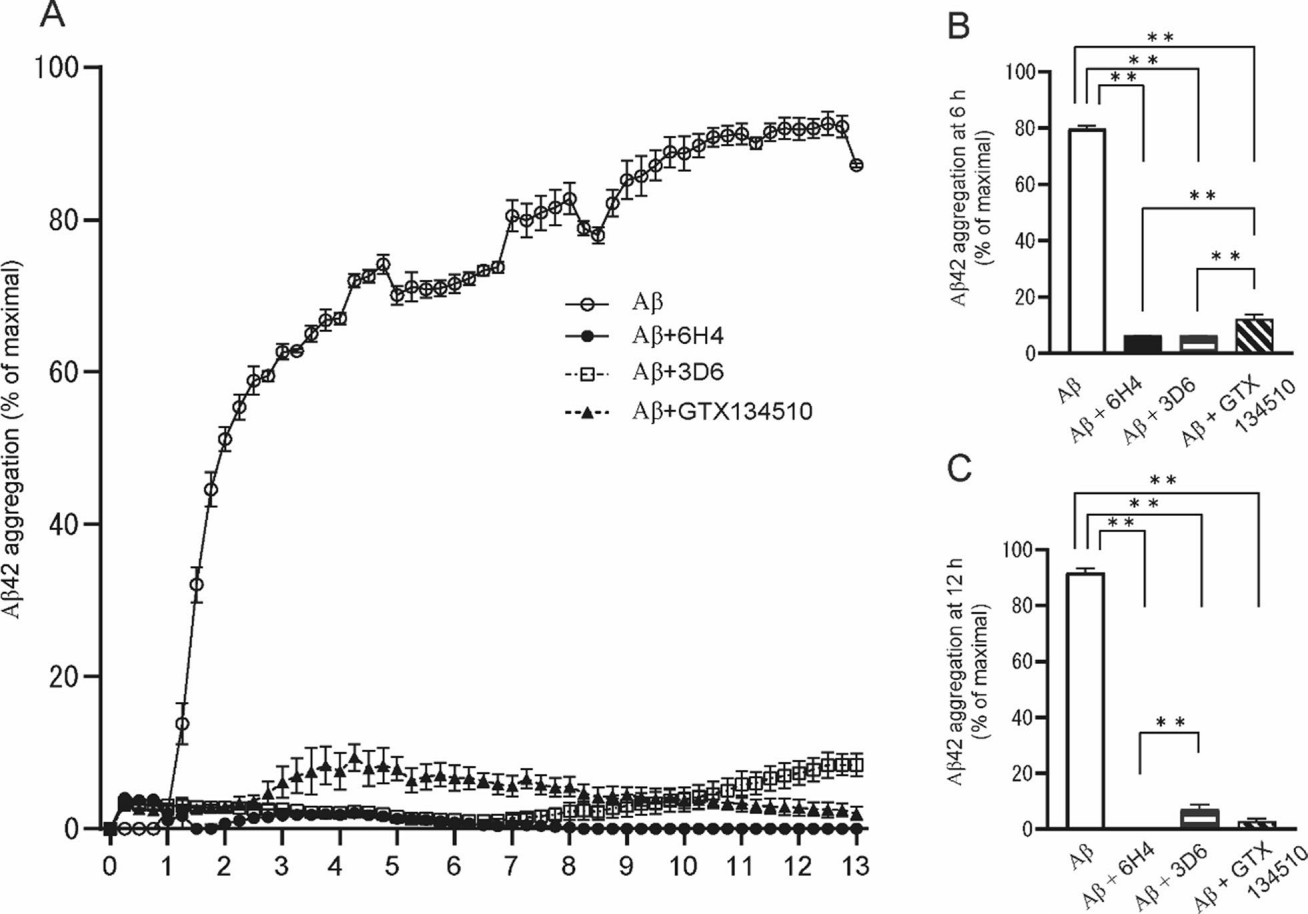
ThT assay of Aβ aggregation. The inhibitory effects of anti-amyloid β (Aβ)O antibody 6H4, anti-Aβ antibody 3D6, and anti-Aβ aggregate-specific antibody GTX134510 on Aβ42 aggregation were evaluated using a thioflavin T (ThT) assay. **A** Aβ alone (white circles) continued to increase the self-aggregation rate even after 1 h of incubation with ThT. In contrast, 6H4 (black circles) showed stable inhibition of Aβ42 aggregation throughout the incubation period. However, compared with 6H4, GTX 134,510 (black triangles) showed a significantly decreased inhibition effect for approximately 3 h of incubation; 3D6 (white squares) showed a significantly decreased inhibition effect after approximately 8 h of incubation. **B** Compared with Aβ alone (white bar) and GTX134510 (hatched bar), 6H4 (black bar) showed a significant inhibition of the Aβ42 aggregation rate after 6 h of incubation. **C** Compared with Aβ alone, 6H4 maintained a 99% inhibition rate on Aβ42 aggregation even after 12 h of incubation; this was significantly higher than the inhibition rate maintained by 3D6 (horizontal striped bar). Values are expressed as mean ± standard error of the mean. Statistical evaluations were performed using one-way or two-way analysis of variance with a Tukey’s post-hoc test. **p < 0.01

The impact of 6H4, an anti-AβO antibody, on Aβ42 aggregation was evaluated using a ThT assay. Aβ42 aggregation was reduced by more than 85% (as compared to the aggregation in the antibody-free control) when any anti-Aβ antibody was added (Fig. 4A). As expected, 6H4 showed long-term, stable inhibition of Aβ42 aggregation throughout the incubation period; however, compared with 6H4, the anti-Aβ fibril-specific antibody GTX 134,510 exerted a significantly lower anti-aggregative effect until approximately 3 h of incubation. Furthermore, compared with 6H4, 3D6 (an anti-Aβ monomer antibody that recognizes amino acid residues 1–5 in Aβ) exerted a significantly lower anti-aggregation effect after approximately 8 h of incubation (Fig. 4A). Anti-AβO antibodies showed a 92.2% reduction (*p* < 0.01, Fig. 4B) in the Aβ42 aggregation rate (as compared with that in the antibody-free control) after 6 h of incubation. The Aβ42 aggregation inhibition effect of anti-AβO antibodies was significantly stronger than that of anti-Aβ fibril-specific antibodies (*p* < 0.01, Fig. 4B). Finally, anti-AβO antibodies achieved a greater than 99% inhibition of Aβ42 aggregation (as compared with that of the antibody-free control) even after 12 h of incubation (*p* < 0.01, Fig. 4C); this inhibitory effect was significantly stronger than that of 3D6 (*p* < 0.01, Fig. 4C).

### Astrocytic endocytotic effects on Fab-amyloid β complexes

**Fig. 5:**
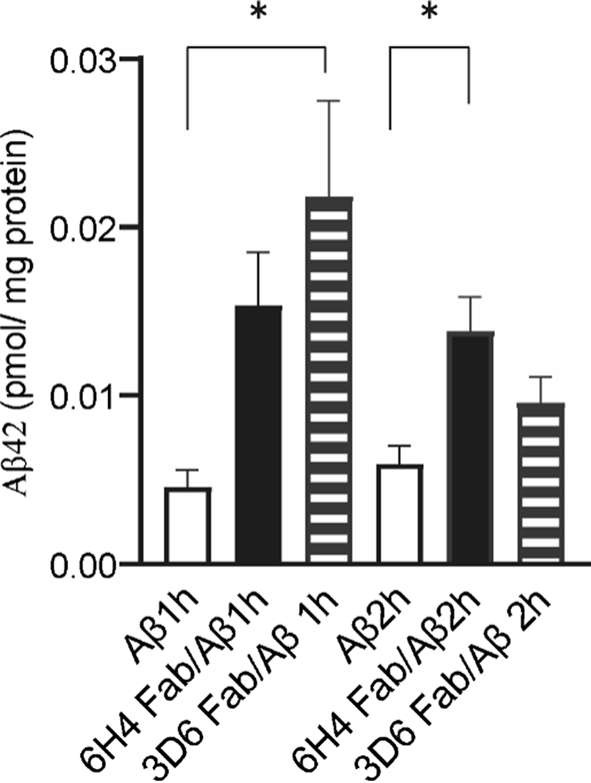
Quantification of intracellular Aβ uptake by fetal rat astrocytes. Astrocytes were plated in a 24-well plate at 20,000 cells per well and cultured for 2 days at 37 ℃. Prior to cellular treatment, 6H4 antibody fragments (Fabs) and 3D6 Fabs were incubated with amyloid β (Aβ)42 to form Fab-antigen complexes at a molar ratio of 1:1. The astrocytes were then incubated for 1 and 2 h with Aβ42s alone (white bar), 6H4 Fab-Aβ42 complexes (black bar), or 3D6 Fab-Aβ42 complexes (horizontal striped bar). Intracellular Aβ42 was quantified using an Aβ amyloid42 enzyme-linked immunosorbent assay kit. A 1-h incubation with the 3D6 Fabs-Aβ42 complexes yielded significantly more (4.7-fold) Aβ42 in the astrocytes than as compared with that obtained by incubation with Aβ alone. Similarly, a 2-h incubation with 6H4 Fab-Aβ42 complexes yielded significantly more (2.3-fold) Aβ42 in the astrocytes as compared with that obtained by incubation with Aβ alone. Statistical evaluations were performed using a one-way analysis of variance with a Tukey’s post-hoc test. *p < 0.05

To investigate how strong of an effect Fab has on Aβ clearance in vitro, we quantified intracellular Aβ uptake by rat astrocytes. After Aβ42 complexed with 6H4 or 3D6 Fabs, those were incubated in the astrocyte culture media for 2 h. A 2.3-fold greater Aβ uptake was observed in astrocytes with the addition of 6H4 Fabs-Aβ42 complexes than that of Aβ alone (*p* < 0.05, Fig. 5). Whereas, adding 3D6-Aβ42 complexes showed significantly greater (4.7-fold) Aβ uptake after 1 h of incubation, which was similar to the findings of a previous study (*p* < 0.05, Fig. 5) [13].

## Discussion

In the present study, the biochemical and pathological efficacies of peripheral administration of anti-AβO 6H4 Fabs encapsulated in PMs to AD model mice, at the age of amyloid plaque deposition in the brain, were investigated. AβO is reported to be significantly toxic when injected into an animal brain or overexpressed in the human brain; it accelerates Aβ seeding [17] and induces pathological AD cascades in the brain thereby impairing the long-term synaptic potentiation of memory circuits [17–19]. Toxic Aβ species, including AβO, toxic Aβ conformers, and N3pE Aβ, were significantly reduced; additionally, one of the histopathological amyloid features of AD, the presence of Aβ plaques with dense cores in the brain, was significantly reduced. The suppression of the progression of pathological processes of AD was supported by the prevention of cognitive-behavioral decline in the spatial memory tests. The results clearly indicate that peripheral administration of anti-AβO 6H4 Fabs in the PMs reduced the amount of toxic Aβ species thereby preventing pathological and behavioral cognitive deterioration. Its efficacy was better compared with that of administration of anti-Aβ 3D6 Fabs encapsulated in the PMs or non-encapsulated 6H4 Fabs, probably because a larger amount of AβO-specific antibodies were delivered into the brain; consequently, they could remove the toxic Aβ species to a greater extent compared with administration of other antibodies or its administration without the PM. The anti-Aβ 3D6 Fabs probably did not remove enough toxic Aβ species, and the non-encapsulated 6H4 Fabs could not pass through the BBB sufficiently.

The pathological AD process and cognitive functions of even the middle-aged mice, which had already developed senile plaques in the brain, were successfully modified by administering 6H4 Fabs in the PMs. The PMs used in the present study have glucose bound to the surface and have the ability to pass through the BBB via glucose transporter 1 on the endothelial cells of the brain arteries. PMs with Fabs were delivered into the brain parenchyma upon elevation of blood glucose levels [12]. The PMs were positively charged and assembled in a solid and stable state with negatively charged Fabs during blood circulation. They were gradually disassembled as the charge-converted Fabs restored their cationic charges based on the acidic environment of endosomes and brain parenchyma, resulting from the loss of ionic interaction and disulfide link breakage [12, 13]. A large amount of intravenously administered Fabs encapsulated in the PMs delivered into the brain was confirmed (see Additional file 1: Fig. S2). The difference in the measurements of Aβ and AβO levels between the 6H4 Fab PM and 6H4 Fab groups was mostly due to the difference in the rates of Fab delivery into the brain. The results in the present study indicate the probability that in previous human clinical trials, one of the causes of the failure in demonstrating the stable efficacy of anti-Aβ antibodies on cognitive function [20] was the insufficient supply of the antibodies in the brain, and, as demonstrated in the present study, the administration of PM-encapsulated effective Fabs can provide clinically sufficient amounts of effective Fabs into the brain, and hopefully prevent the progression of AD pathology.

Another important factor in the present study was that anti-AβO Fabs could reduce various toxic Aβ species, including AβOs, toxic Aβ conformers, and N3pE Aβs. We previously demonstrated that anti-AβO antibodies lead AβOs to form nonfibrillar amorphous structures and play an important chaperone-like role in the Aβ aggregation pathway and allow Aβ to exist in a non-toxic state using a ThT assay and electron microscopy [21]. The present study verified the inhibitory effect on Aβ42 aggregation using a ThT assay and found that only 6H4 showed a long-lasting inhibitory effect on Aβ aggregation, such as primary nucleation and seeds (Fig. 4A). The anti-Aβ 3D6 antibody used in this study reportedly prevents Aβ plaque formation, although it does not remove existing plaques in the brain [22]. 3D6 also exerted an inhibitory effect on Aβ aggregation, although it was probably insufficiently effective in inhibiting the formation of highly aggregated structures such as Aβ fibrils (Fig. 4C). Contrary to 3D6, GTX 134510 may exert an anti-aggregatory effect on Aβ42 hyperaggregation, which was observed after prolonged incubation (Fig. 4A and C). Although aducanumab binds to a linear epitope of three to seven amino acids of the Aβ peptide and mainly binds to aggregates and oligomers, it has very low affinity for N3pE Aβs [23]. Conversely, the N3pE Aβ-specific antibody mE8 (donanemab) reportedly improves cognitive functions in patients with early AD [24, 25] and removes existing Aβ plaques [24]. The N3pE Aβ production pathway is activated by age-dependent and AD pathology-induced downregulation of neprilysin, an age-dependent endopeptidase for Aβ in the brain [26]. Glutaminyl cyclase, which cleaves and cyclizes the amino-terminal Aβ [6], increases with the progression of AD [6]. The aggregation and oligomerization rates of N3pE Aβs are faster than those of unmodified Aβs [5, 27]. Interestingly, the present study revealed a significant reduction in the number of Aβ plaques with dense cores, whereas diffuse plaques, which lacked dense cores, were observed in the brains of the 6H4 Fabs PM group. Biochemical analyses revealed a significant reduction in the levels of toxic Aβ species. The results indicated that the administration of 6H4 Fabs in the PMs may serve as a treatment strategy to reduce these toxic Aβ species concurrently and to modify the AD pathologic process even after the initiation of pathological AD cascades. In addition, the present study confirmed the role of 6H4 in preventing the spread of AβOs: 6H4 Fab PM administration decreased AβOs in the cortical area and hippocampus (Additional file 1: Fig. S4). Using molecular dynamics simulations, new candidates for AD treatment that can enable the degradation of AβOs or protofibrils and reduced neurotoxicity [28] have been produced, with some molecules binding to Aβ sites where Aβ can be destabilized [29, 30].

Regarding the lack of Fc antibody sites, administration of an anti-AβO 72D9 full-body antibody, which had Fc sites, reduced Aβ plaques without microglial responses in the brains of AD model mice [14]. The Fc site of the antibody mediates the cellular uptake of immune complexes and widely activates them to phagocytose various pathogenic degradation products. In contrast, a fragment antibody that lacks an Fc site, such as the one used in the present study, reportedly mediates another clearance system for Aβ in vivo [31]. 3D6 Fabs in the PMs activate astrocytes to uptake immune complexes of Aβs and Fabs [13]. Astrocytes are involved in soluble Aβ clearance in the brain by facilitating intracellular degradation and promoting expulsion into the circulatory system via the BBB or “glymphatic” system [13]. Moreover, Fabs may have the ability to modulate the pathway via astrocytic endocytosis. Indeed, Aβ42 uptake was significantly higher in astrocytes incubated with the 6H4 Fab-Aβ complexes than in astrocytes incubated without the antibodies (Fig. 5). Similar to our previous study [13], astrocytes incubated with 3D6 Fab-Aβ complexes and 6H4 Fab-Aβ complexes showed significantly higher amounts of intracellular Aβ after 1 and 2 h of incubation, respectively (Fig. 5). The reduction of Aβ uptake in astrocytes incubated with 3D6 Fab-Aβ complexes was probably due to the cytotoxicity of endocytotic Aβ because a significantly increased lactate dehydrogenase activity was observed after 2 h only in the culture medium (Additional file 1: Fig. S5). In our previous study, the uptake assay was performed in experiments using culture media with serum, which partly protected the astrocytes from Aβ cytotoxicity. The results of the present study indicate that 6H4 Fabs may have the ability to inhibit Aβ aggregation and bind to soluble Aβ to modulate astrocytic Aβ uptake, thereby facilitating Aβ clearance with less cytotoxicity. Moreover, a possible advantage of administering Fab is that ARIA can be avoided because of the lack of Fc gamma receptor activation [29].

The present study had some limitations. First, under the coronavirus disease 2019 pandemic, the Japanese government was restricted to perform usual experimental activities in Japanese institutes, which caused a delay of a few months in the administration experiments, and the types of behavioral tests that could be analyzed were restricted. Second, regarding the mechanisms of Aβ excretion in the brain, it was difficult to identify whether degraded Aβs was excreted via the glymphatic system. Third, the availability of AD model mice was limited in this study due to the high incidence of sudden death among males after weaning, although female mice (who generally have higher survival rates) were used to counter this issue, multiple factors (such as estrous hormone disorders) may have led to differences in their behaviors, which in turn may have confounded our findings. Finally, separate quantification of toxic Aβ species in the brain parenchyma and in the endothelial cells was technically difficult.

## Conclusions

Larger amounts of anti-AβO 6H4 Fabs encapsulated in PMs pass through the BBB of AD model mice and are released inside the brain. The Fabs recognized various toxic Aβ species concurrently and reduced their amount, inhibited Aβ-induced AD pathological processes, and alleviated cognitive decline.

## Methods

### Study design

The efficacy of 10-week peripheral administration of newly developed anti-Aβ oligomer encapsulated in PMs in an AD mouse model, was evaluated and compared with PBS administrated to control mice. The mice brains were examined biochemically and immunohistochemically for AD pathology markers, and behavioral tests were performed.

### Antibodies and Fabs encapsulated in the PMs

The 6H4 antibody was prepared as described previously [14] and purified for insertion into human/mouse chimeras of immunoglobulin (Ig)G1 and mouse monoclonal IgG2b. A humanized 3D6 antibody (Bapineuzumab, IgG1) recognizing amino acid sequences from 1–5 of Aβ was also generated. Monoclonal 82E1 antibody (IgG1, #10323, Immuno-Biological Laboratories Co., Ltd, Gunma, Japan) recognizing Aβ1-16 and anti-human Aβ N3pE rabbit antibodies (IgG, #18591, Immuno-Biological Laboratories Co.) recognizing N3pE Aβ3-42 and -40 were purchased. Synthetic Aβ1-40 monomers and 5-carboxytetramethylrhodamine (TAMRA)-conjugated Aβ1-40 monomers were purchased from AnaSpec, Inc. (Fremont, California, USA). Fabs were prepared as described previously [13]. The internalization system was composed of pH/redox-responsive PM composed of cationic disulfide cross-linked polymers complexed with charge-converted anionic Fabs, which can restore their charge and bioactivity under acidic conditions [12, 13]. By setting up a glucose functionalization targeting the glucose transporter 1, the ability to cross the BBB under specific glycemic control conditions was conferred on the PM with glucose conjugating to 25% of the constituent polymer chains [13].

### Model animals

Heterozygous APPswe/PS1dE9 mice (“B6.Cg Tg [PPswe, PSEN1dE9] 85Dbo/ Mmjax,” MMRRC Stock No.34832-JAX) (The Jackson Laboratory, Bar Harbor, ME, USA) having a double mutation (one in the *APP* gene [Swedish mutations K595N/M596L; APPswe) and one in human presenilin 1 [deletion of exon 9; PS1dE9) and wild-type (WT) littermates mice were obtained by breeding at the laboratory animal center. The heterozygous APPswe/PS1dE9 mice are maintained and used for research; they are usually obtained by mating female C57BL/6J mice with heterozygous male APPswe/PS1dE9 mice.

At 37 weeks of age, the mice were pretreated as previously described [13] and were classified into five groups according to the materials administered as follows:

(i) PBS group: seven APPswe/PS1dE9 female mice were administered PBS;
(ii) 6H4 Fab PM group: nine APPswe/PS1dE9 female mice were administered 6H4 Fab-PMs;
(iii) 3D6 Fab PM group: eight APPswe/PS1dE9 female mice were administered 3D6 Fab-PMs;
(iv) 6H4 Fab group: five APPswe/PS1dE9 female mice administered with 6H4 Fabs; and
(v) control group: five male WT mice.

Then, 1.8 mg Fabs/kg body weight/week was peripherally administered via the caudal vein every week for 10 weeks until 47 weeks of age. Cognitive function was evaluated at 48 weeks of age using the Morris water maze (MWM) and Y-maze tests. The mice were sacrificed after the termination of the behavioral tests for biochemical and histopathological analyses.

All mice had free access to water and were fed a CE-2 diet (CLEA Japan, Inc., Tokyo, Japan) until the end of the experiments. The mice were maintained on a 12 h light/dark cycle in a controlled environment.

### Preparation of Fabs encapsulated in the PMs

Fabs (6H4 and 3D6) PMs were assembled through an ionic interaction between a cationic poly(ethylene-glycol)-poly(l-lysine) (PEG-PLL) block copolymer and a charge-converted anionic Fab antibody fragment. Cationic PEG-PLL block copolymer and a charge-converted anionic Fab antibody fragment were used to formulate Fabs (6H4, 3D6) PMs through ionic interaction. Charge converted Fabs were obtained by coupling positively charged primary amino groups with citraconic anhydride (Cit) through an amidation reaction [13, 32, 33] This reaction produces a negatively charged Cit-Fab with an increased number of carboxylate groups. The Fab charge conversion ratio (60% of amino groups per Fab occupied by Cit) was adopted from a previous report [13]; it reportedly ensures sufficient PM formation. This method of charge-conversion was adopted because the original primary amino groups can be recovered in low pH environments through the removal of the citraconic amide bonds, which reverses the charge conversion and recovers the proper antigen binding ability of Fabs [13, 32, 33]. PEG-PLL copolymers were capped with attaching either a methoxy (termed MeO-PEG-PLL) or a glucopyranos-6-O-yl (Gluc-PEG-PLL) group to the distal end of the PEG segment to modulate PM glucose decoration prior to complexation with the Cit-Fab. To facilitate disulfide cross-linking in the PM core, lysine residues in the polymers were partially derivatized with 3-(2-pyridyldithio)propionate (PDP) groups (this led to PEG-PLL-PDP formation) [13, 34], and subsequent enhancement of PM stability (Additional file 2: Methods S1–S3 and see Additional file 3: Table S1). The PDP modification ratio of Gluc-PEG-PLL and MeO-PEG-PLL in the present study was 30% (i.e., 30% of lysine residues modified for MeO-PEG-PLL and for Gluc-PEG-PLL), which was optimized in previous studies [13].

### Protein isolation

Mice were anesthetized and perfused with PBS, and the right cerebral hemispheres of the brain were immediately frozen in liquid nitrogen for biochemical experiments. The left cerebral hemispheres were embedded in optimal cutting temperature compound (Sakura Finetek Japan Co., Ltd., Tokyo, Japan) for immunohistochemistry. The samples were stored at − 80 ℃. The brains were homogenized for biochemical experiments in buffer containing 50 mM Tris-HCl (pH 7.4), 150 mM NaCl, and Halt™ Protease Inhibitor Cocktail, EDTA-free (Thermo Fisher Scientific, Waltham, MA, USA) using a microhomogenizer (Nippon Genetics Co., Ltd., Tokyo, Japan). For the dot-blot assay, the supernatant after centrifugation of the homogenate at 13,000*g* for 15 min at 4 °C was used. For ELISA, the homogenates were centrifuged at 100,000*g* for 30 min at 4 ℃, and the supernatant was used as the tris-buffered saline (TBS)-soluble fraction. The precipitates were dissolved in 6 M GuHCl and treated with an ultrasonicator (UH-150; SMT Co., Ltd., Tokyo, Japan) followed by an ultrasonic bath (USM-1; AS ONE Corporation, Osaka, Japan) for 15 min. These were centrifuged at 21,000*g* for 20 min to obtain the supernatant (TBS-insoluble fraction). Each fraction was processed using a Pierce™ BCA Protein Assay Kit (Thermo Fisher Scientific); the absorbance was measured at 562 nm using an Infinite M1000 Pro microplate reader (Tecan Group Ltd., Männedorf, Switzerland) to determine the protein concentration.

#### Enzyme-linked immunosorbent assay

Each indicator in the brain was quantified according to the manufacturer’s protocols using the following: Aβ N3pE40 (IBL #27418 human Aβ [N3pE-40] assay kit, Gunma, Japan), Aβ N3pE42 (IBL #27716 human Aβ [N3pE-42] assay kit), Aβ40 (human/rat Aβ [40] ELISA kit) (FUJIFILM Wako Pure Chemical Corporation, Osaka, Japan), Aβ42 (human/rat β amyloid (42) ELISA kit) (FUJIFILM Wako Pure Chemical Corporation), and a toxic conformer (IBL #27709 human Aβ toxic oligomer assay kit). Absorbance was measured using an Infinite M1000 Pro (Tecan Group Ltd.) and for ELISA.

#### Quantitative dot-blot assay

To quantify Aβ species in the mouse brain, the supernatants of brain homogenates were spotted three times for each μL on nitrocellulose membranes (0.2 μm pore size, Bio-Rad Laboratories, Inc., Hercules, California, USA). These membranes were blocked with 5% skimmed milk in TBS (0.1 M Tris–HCl [pH 7.6], 0.15 M NaCl) for 1 h and then incubated with an anti-AβO antibody (mouse monoclonal 6H4) for 1 h at 4 ℃, followed by horseradish peroxidase-labeled anti-mouse IgG antibody. A standard consisting of Aβ 1–40 and TAMRA labeled Aβ1-40 mixture was used to prepare the calibration curve for AβO. The toxic conformer standards used were the ELISA standards of the toxic conformers (IBL #27709 human Aβ toxic oligomer assay kit). Chemiluminescent signals were detected with a ChemiDoc Touch (Bio-Rad Laboratories, Inc.) using ECL Prime Western Blotting Reagent (GE Healthcare Chicago, IL, USA). The signal intensity was quantified using Image Lab software (Bio-Rad Laboratories, Inc.).

### Immunohistochemistry

#### Immunofluorescence staining

Frozen brain sections were fixed with Mildform 10 N (FUJIFILM Wako Pure Chemical Corporation) and blocked with 5% normal goat serum in PBS for 1 h and then incubated with anti-AβO antibodies (6H4) overnight at 4 °C. The sections were then incubated with goat-antimouse secondary antibodies (Alexa Fluor 647; A-21242; 1:500; Thermo Fisher Scientific) for 1 h at 26 °C. Next, the sections were incubated with filtered 0.002% thioflavin S (Merck KGaA, Darmstadt, Germany) solution dissolved in PBS for 10 min. The sections were then mounted in the presence of Fluoromount/Plus (Diagnostic BioSystems, Pleasanton, CA, USA) and examined using a TCS SP8 confocal laser microscope (Leica, Wetzlar, Germany). For quantitative analysis, the signal intensity (%) per ROI of immunoreaction and thioflavin S staining was determined using Leica Application Suite X software (Leica). The secondary auditory and secondary visual cortices and adjacent areas were selected as ROIs (762 × 762 μm).

#### Immunohistochemistry

Frozen sections fixed with Mildform 10N were pretreated with 0.3% H_2_O_2_ in MeOH to block endogenous peroxidases, then blocked with 2.5% normal horse serum (Vector Laboratories, Inc., Newark, CA, USA) for 1 h. Sections were stained with anti-human Aβ (N3pE) Rabbit IgG Affinity Purify (1:100) (IBL #18591) or anti-human Aβ (82E1) mouse IgG MoAb (1:100) (IBL #10323), overnight at 4 °C. The sections were then incubated with Immuno PRESS Reagent, Anti-rabbit IgG (Vector Laboratories, MP-7401), or Immuno PRESS Reagent, Anti-Mouse IgG (Vector Laboratories, MP-7402) for 1 h at room temperature. The color was developed using the Dako Liquid DAB (3,3-diaminobenzidine) + Substrate Chromogen System (Agilent Technologies, Inc., Santa Clara, CA, USA) and examined using an all-in-one fluorescence microscope BZ-X700 (Keyence Corporation, Osaka, Japan) as a phase-contrast microscope. For quantitative analysis, the positive areas in immunostaining (%) per ROI (1225 × 1225 μm) or plaque diameter were determined using a BZ-X analyzer (Keyence). The diameter was calculated as the mean of 20 randomly selected plaques, each within the multiple ROIs of each mouse.

### Behavioral test

The MWM and Y-maze tests were performed as previously described with minor modifications [35, 36]. The hidden platform test for MWM was performed according to the manufacturer’s protocol [35]. Briefly, mice were placed in a gray circular plastic pool with a diameter of 120 cm in a room where visual cues were placed outside the pool. The 10-cm-in-diameter hidden platform was located 1.0 cm below the water surface as a goal. The trials were video recorded, and the data were analyzed using the EthoVision XT system (Noldus, Wageningen, Netherlands). Four trials per day were performed for all mice for five days during the learning phase. Each mouse underwent a maximum of a 60-s trial to reach the hidden platform. When a mouse reached the goal, the investigators let it stay there for 20 s and then returned it to a resting cage. Otherwise, the investigators removed the mouse from the water to the platform for 20 s. One day after the learning trial, a hidden platform test was conducted for three consecutive days. During the hidden platform test, the mice were allowed to swim freely for a maximum of 180 s, and the time required to reach the platform was measured. A probe test was also performed to calculate the percentage of time the mice stayed in the restricted areas, which were virtually separated areas in the circular pool defined as start, platform, side 1, and side 2, for up to 180 s of swimming time.

The Y-maze task was performed to evaluate short-term and working spatial memories [36]. Mice were placed on the Y-maze consisting of three equally spaced arms (120° apart, 40 cm long, and 10 cm wide) made of gray plastic plates and were allowed to walk freely for 4 min to familiarize itself during the training period. One day after training, mice were placed in one of the arms and allowed to explore the maze for 4 min, during which the sequence and number of arm entries were recorded using the EthoVision XT video system. The spontaneous alternation rate (%) for each group was calculated and compared using the following formula:

$$\frac{number\,of\,spontaneous\,alternations}{total\,number\,of\,arm\,entries - 2} \times 100$$

### Thioflavin T assay

The impact of 6H4 on Aβ42 aggregation was evaluated using a ThT assay as described previously [13, 21]. Briefly, Aβ42 (Merck, AG968) was monomerized with hexasuppleisopropanol and dissolved in 1% NH_4_OH. Full-body 6H4, 3D6, and anti-Aβ aggregate-specific antibody GTX134510 (GeneTex, Inc., Irvine, CA, USA) and Aβ42 solution (final concentration: 12.5 μM) were diluted in PBS; the molar ratios were 1:10 and 0:1 for the aforementioned antibodies and Aβ42, respectively. ThT (FUJIFILM Wako Pure Chemical Corporation) in 50 mM glycine–NaOH was added to the Aβ42 and Aβ42-antibody mixtures at a final concentration of 10 μM. Aβ42 aggregation was monitored every 15 min for 13 h of shaking at 37 °C using an Infinite M1000 Pro microplate reader (Tecan Group Ltd) at excitation and emission wavelengths of 440 and 480 nm, respectively.

### Astrocyte preparation

We prepared primary cultured astrocytes from cerebral cortices of rats as described previously [13, 37]. Briefly, mouse embryos were removed from pregnant Crl:CD(SD) rats (Oriental Yeast Co., Ltd., Tokyo, Japan) under anesthesia on embryonic day 19. After the cerebral cortices of the fetal rats were dissected, the meninges were removed in cold Hanks' Balanced Salt Solution (FUJIFILM Wako Pure Chemical Corporation) under a dissection microscope (NIKON CORPORATION, Tokyo, Japan). The cortices were then minced in Dulbecco's Modified Eagle Medium (DMEM; FUJIFILM Wako Pure Chemical Corporation) containing 10% fetal bovine serum (Thermo Fisher Scientific), 100 units/mL of penicillin, and 100 µg/mL of streptomycin (Thermo Fisher Scientific); this was achieved by passing the tissue through an 18-gauge sterile syringe (Terumo Corporation, Tokyo, Japan). The dissociated cell suspensions were plated with a T-75 culture flask (Thermo Fisher Scientific). After 10 days, the astrocytes were confluent with overlaying microglia cells. To eliminate residual microglia, 10-day-old flasks were agitated at 180 rpm for 6 h. The astrocytes were detached using 2.5% trypsin/EDTA (Nacalai tesque, INC., Kyoto, Japan) and replated onto a T-75 culture flasks for experimental or continued expansion. Positivity for the astrocyte-specific glial fibrillary acidic protein marker (Merck KGaA, G9269) and negativity for the microglia-specific ionized calcium-binding adapter molecule 1 marker (FUJIFILM Wako Pure Chemical Corporation, 019-19741) in the isolated astrocytes were confirmed using immunofluorescence staining.

### Aβ42 uptake by astrocytes

Astrocytes from fetal rat cerebrums were plated in a 24-well plate at 20,000 cells per well and cultured with DMEM containing 10% fetal bovine serum for 2 days at 37 ℃. Prior to cellular treatment, 6H4 Fab and 3D6 Fab were complexed with Aβ42 in PBS for 18 h at 4 °C at a molar ratio of 1:1 (0.05 pmol of each), as described previously [13]. The cells were then incubated for 1 and 2 h with Aβ42s alone, 6H4 Fab/Aβ42 complexes, and 3D6 Fab/Aβ42 complexes. Aβ42s were prepared at a concentration of 100 pM in 500 μL serum-free DMEM. After incubation, the medium was collected for cytotoxic lactate dehydrogenase assays (see Additional file 1: Fig. S5), and the cells were washed with PBS and lysed with passive lysis buffer (#E194A, Promega Corporation, Fitchburg, WI, USA) to measure the Aβ42 uptake. Aβ42 was quantified using a β amyloid (42) ELISA kit (#290-62601, Fujifilm Wako Pure Chemicals Co.). Protein concentrations of the lysates were measured using the Pierce™ BCA Protein Assay Kit and an Infinite M1000 Pro microplate reader.

### Statistical analysis

Results are expressed as the mean ± standard error of the mean. The probability of statistical differences between experimental groups was determined by analysis of variance followed by Tukey or Dunnett’s post hoc test for multiple comparisons using GraphPad Prism software (GraphPad Software, La Jolla, CA, USA).

## Supplementary Information

**Additional file 1.** Quantitative supplemental experiments.** Fig. S1.** Quantitative measurement of soluble Aβ species and specificity of 6H4 antibody to the toxic conformers. **Fig. S2.** Quantitative measurement of the rate of the Fabs passing through the blood–brain barrier of the mice. **Fig. S3.** Dot-blot assay of AβO. **Fig. S4.** Quantitatively evaluation of immune-positivity of Aβs in the brain sections. **Fig. S5.** Evaluation of astrocyte cytotoxicity by LDH activity.

**Additional file 2.** Detailed Fabs and PM preparation methods. **Methods S1.** Preparation of Citraconic Anhydride Modified Fabs (Cit-Fabs). **Methods S2.** Preparation and Characterization of Fabs PMs. **Methods S3. **Preparation of fluorescently labeled 6H4 and 3D6 Fabs.

**Additional file 3.** Tables of PMs and data availability of the study. **Table S1.** DLS Analysis of Fabs Encapsulated in the PMs. **Table S2.** Quantitative values of the results.

## Data Availability

All data generated or analyzed during this study are included in this published article and its Additional files. The quantitative results obtained are summarized in (see Additional file 3: Table S2).

## Abbreviations

AD: Alzheimer’s disease
Aβ: Amyloid β
AβO: Amyloid β oligomer
ARIA: Amyloid-related imaging abnormalities
BBB: Blood–brain barrier
Cit: Citraconic anhydride
DMEM: Dulbecco's Modified Eagle Medium
ELISA: Enzyme-linked immunosorbent assay
Fab: Antibody fragment
Fc: Fragment crystallizable
Gluc: Glucopyranos-6-O-yl
Ig: Immunoglobulin
MeO: Methoxy
MWM: Morris water maze
N3pE Aβ: Pyroglutamated amyloid β
PBS: Phosphate-buffered saline
PDP: 3-(2-Pyridyldithio)propionate
PEG: Poly(ethylene-glycol)
PLL: Poly(l-lysine)
PM: Polymeric nanomicelle
PS1dE9: Presenilin 1 with deletion of exon 9
ROI: Region of interest
TAMRA: 5-Carboxytetramethylrhodamine
ThT: Thioflavin
TBS: Tris-buffered saline
WT: Wild type
