# Supplementary material for: Peripheral administration of nanomicelle-encapsulated anti-Aβ oligomer fragment antibody reduces various toxic Aβ species in the brain
Source: J Nanobiotechnology. 2023 Jan 31;21:36. doi: 10.1186/s12951-023-01772-y (PMC9888736; doi:10.1186/s12951-023-01772-y)
Supplement: Supplementary file 1 — Additional file 1. Quantitative supplemental experiments. Fig. S1. Quantitative measurement of soluble Aβ species and specificity of 6H4 antibody to the toxic conformers. Fig. S2. Quantitative measurement of the rate of the Fabs passing through the blood–brain barrier of the mice. Fig. S3. Dot-blot assay of AβO. Fig. S4. Quantitatively evaluation of immune-positivity of Aβs in the brain sections. Fig. S5. Evaluation of astrocyte cytotoxicity by LDH activity. [file 12951_2023_1772_MOESM1_ESM.pdf]

**Additional file 1:**

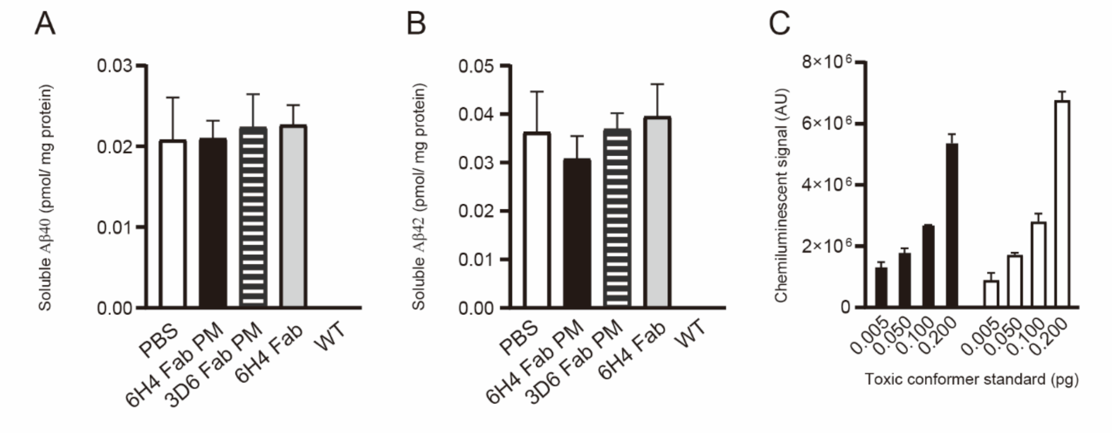


**Additional file 1: Fig. S1. Quantitative measurement of soluble Aβ species and specificity of 6H4 antibody to toxic conformers**

Tris-buffered saline-soluble Aβ40 **(A)** and Aβ42 **(B)** in brain homogenates of Alzheimer's disease model mice were quantified using enzyme-linked immunosorbent assay. There was no significant difference in the amounts of Aβ40 or Aβ42 among the groups**.**

Diluted standards of the toxic conformers were incubated with antitoxic conformer 11A1 antibodies (white bars) and 6H4 antibodies (black bars). After incubation with the secondary horseradish peroxidase-labeled anti-mouse immunoglobulin G antibody, chemiluminescent signals were measured. Dose-dependent specificity was identified for both antibodies **(C)**. The calibration curve of toxic conformers (μM) and Aβ oligomers are : $Y(11A1) = -1.03X^2 + 32.21X - 245.58$ and $Y(6H4) = -2.9X^2 + 88.67X - 670.66$, respectively.

Values are expressed as the mean ± standard error of the mean. Two-way analysis of variance with Dunnett's post hoc test was performed.

Aβ, amyloid β; Fab, antibody fragment; PBS, phosphate-buffered saline; PM, polymeric nanomicelle; WT, wild type

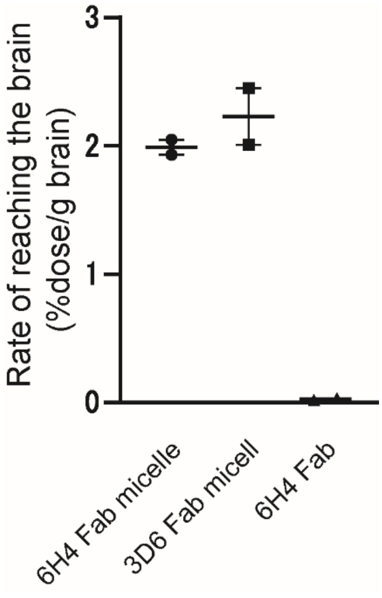


**Additional file 1: Fig. S2. Quantitative measurement of the rate of Fabs passing through the blood-brain barriers of mice**

The 6H4 and 3D6 antibody fragments (Fabs) in the polymeric nanomicelles (PMs) and 6H4 Fabs without PMs were fluorescently labeled and injected intravenously (Fab = 1.5 mg/kg) into 6 week-old, female, C57BL/6J mice (n = 2 in each group). The 6H4 (black squares) and 3D6 (black circles) rates of Fabs encapsulated in the PMs in the brains of wild-type mice were measured for fluorescence intensity of the labeled Alexa647 using a microplate reader (Tecan Group Ltd.), as we previously described [13]. Both 6H4 and 3D6 Fabs in mice brains were approximately 80 times larger than 6H4 Fabs without PMs.

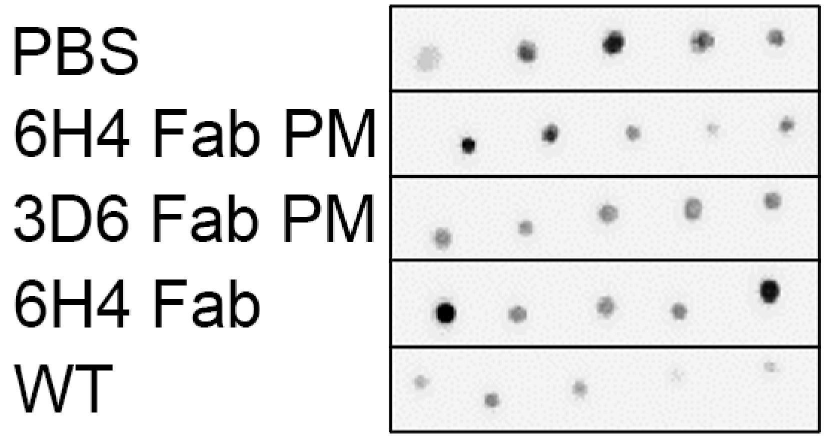


**Additional file 1: Fig. S3. Dot-blot assay of AβO**

The dot-blot of the brain homogenates of Alzheimer's disease model mice that were administered 6H4 ands 3D6 Fabs in the PMs; 6H4 Fabs are demonstrated. The amounts of AβO in the brain homogenates was quantified by calculating the average signal densities of the dots and was compared among the groups (Fig. 1C). The density of the 6H4 Fab PM group was 65% lower than that of the PBS group.

AβO, amyloid β oligomer; Fab, antibody fragment; PBS, phosphate-buffered saline; PM, polymeric nanomicelle; WT, wild type

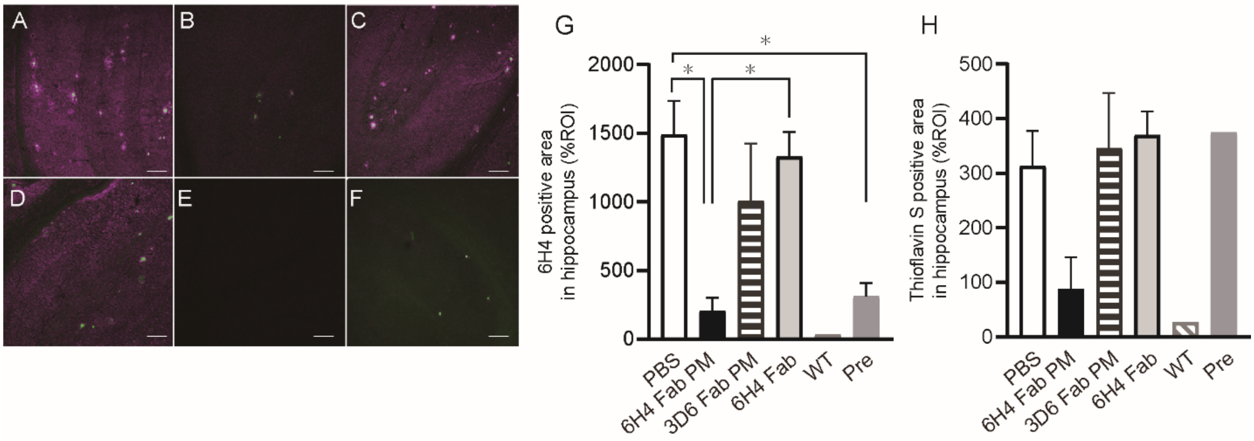


**Additional file 1: Fig. S4. Quantitative evaluation of immune-positivity of Aβs in brain sections**

Images of the immunofluorescent positive signals for anti-AβO 6H4 antibody (magenta) and thioflavin S (green) in AD mice treated with PBS **(A)**, 6H4 Fab PMs **(B)**, 3D6 Fab PM **(C)**, and 6H4 Fab **(D)**, and WT mice **(E)**. Stained images of untreated AD mice at the beginning of the experiment are also shown **(F)**. Quantitative analysis of the %ROI of 6H4 antibody **(G)** and thioflavin S **(H).** The positive signals observed in AD mice in panels A–D showed a significant decrease in the number of 6H4 positive signals in the 6H4 Fab PM group (black bar) compared with that in the PBS group (white bar) and a decreasing trend in thioflavin S. Scale bar=100 μm. One-way analysis of variance with Tukey's post-hoc test was performed. Values are expressed as the mean ± standard error of the mean. *$p < 0.05$.

AβO, amyloid β oligomer; AD, Alzheimer's disease; Fab, antibody fragment; PBS, phosphate-buffered saline; PM, polymeric nanomicelle; ROI, region of interest; WT, wild type

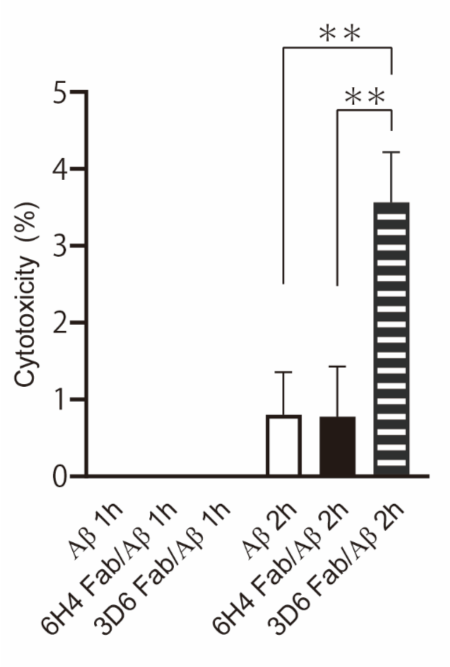


**Additional file 1: Fig. S5. Evaluation of astrocyte cytotoxicity by LDH activity**

Astrocytes from a fetus rat cerebrum incubated for 1 and 2 hours with Aβ42s alone (white bar), 6H4 Fab-Aβ42 complexes (black bar), or 3D6 Fab/Aβ42 complexes (horizontal striped bar). After incubation, the medium was collected and cytotoxic lactate dehydrogenase (LDH) activity was quantified using a Cytotoxicity LDH Assay Kit-WST (DOJINDO LABORATORIES, Kumamoto, Japan). Compared with that in the other groups, significantly higher LDH activity was only observed in the culture medium 2 h after the 3D6 Fab/Aβ complex. Values are expressed as the mean ± standard error of the mean. One-way analysis of variance with Tukey's post hoc test was performed. ** $p < 0.01$

Aβ, amyloid β; Fab, antibody fragment
