# Supplementary material for: Peripheral administration of nanomicelle-encapsulated anti-Aβ oligomer fragment antibody reduces various toxic Aβ species in the brain
Source: J Nanobiotechnology. 2023 Jan 31;21:36. doi: 10.1186/s12951-023-01772-y (PMC9888736; doi:10.1186/s12951-023-01772-y)
Supplement: Supplementary file 2 — Additional file 2. Detailed Fabs and PM preparation methods. Methods S1. Preparation of Citraconic Anhydride Modified Fabs (Cit-Fabs). Methods S2. Preparation and Characterization of Fabs PMs. Methods S3. Preparation of fluorescently labeled 6H4 and 3D6 Fabs. [file 12951_2023_1772_MOESM2_ESM.docx]

**Additional file 2**

**Methods 1. Preparation of Citraconic Anhydride-Modified Fabs**

Citraconic anhydride-modified antibody fragments (Cit-Fabs) were prepared as follows. First, the Fab solution (0.5 mg/mL) was prepared by diluting with phosphate-buffered saline (0.1 M, pH = 8.5). Citraconic anhydride (which contains five times the molar number of the amino groups to be modified in Fabs) was slowly added to the solution; the solution was then stirred for 1 h at room temperature. The derivatives were purified by ultrafiltration (Vivaspin 6, molecular weight cutoff: 30 kDa, 10000 rpm) three times with phosphate-buffered solution (10 mM, pH = 7.4). To calculate the degree of modified amines, the number of unmodified amines was quantified using fluorescamine. Fab derivatives (6 μL, 0.4 mg/mL; 10 mM phosphate-buffered solution) were incubated with fluorescamine (2 μL, 3 mg/mL; dimethylformamide) for 10 min at room temperature, and the fluorescent signal was measured using an ND-3300 fluorospectrometer (Nanodrop, Wilmington, DE, USA).

**Methods 2. Preparation and Characterization of Fabs PMs**

Antibody fragments (Fabs) encapsulated in the polymeric nanomicelles (PMs) with 25% glucose decoration were prepared as follows. First, glucopyranos-6-O-yl (Gluc)-poly(ethylene-glycol)-poly(l-lysine) (PEG-PLL)-3-(2-pyridyldithio) propionate (PDP; 0.5 mg/mL) and methoxy (MeO)-PEG-PLL-PDP (0.5 mg/mL) were mixed at a 4:1 ratio to prepare PMs decorated with glucose on 25% of their surface. The polymers were incubated in a 200 mM dithiothreitol solution at 37 °C to break down the disulfide bonds within the PDP groups, leading to the production of free thiols in the polymers. The derivative solution was subsequently purified through ultrafiltration (Vivaspin 6, molecular weight cutoff: 10 K Da) with phosphate-buffered solution (10 mM, pH 7.4). Then, citraconic anhydride-modified-Fabs (0.4 mg/mL) were mixed with the polymer solution (0.5 mg/mL) at a feeding positive/negative charge ratio of 2:1 in phosphate-buffered solution (10 mM, pH 7.4). The resulting solution was incubated for 24 h at room temperature to form disulfide cross-linking in the core of the PMs. For characterization of the resulting Fab PMs, size distribution and z potential were evaluated via dynamic light scattering using a Zetasizer Nano ZS90 (Malvern Instruments Ltd., Worcestershire, UK) at 25 °C (Supplementary Table 1).

**Methods 3. Preparation of fluorescently labelled 6H4 and 3D6 Fabs**

Fluorescent dye (Alexa647)-labeled antibody fragments (Fabs; 6H4 and 3D6) are prepared by first adding 10 mg/mL of Alexa647-NHS (dissolved in dimethyl sulfoxide) to the Fab solutions (0.1 M phosphate-buffered solution, pH = 8.5) at a dye:Fab molar ratio of 10:1. The mixture is stirred at room temperature for 1 h and purified by ultrafiltration (Vivaspin 6; molecular weight cutoff: 30K Da, 10,000 rpm) three times with 10 mM phosphate-buffered solution (pH 7.4). The molar ratio of Alexa647 to Fab of Alexa647-Fab was determined from the ratio of Alexa64- and Fab-derived absorbance, which indicated Alexa647:Fab of 1.35 and 1.31 for 6H4 and 3D6, respectively.
