# Supplementary material for: Peripheral administration of nanomicelle-encapsulated anti-Aβ oligomer fragment antibody reduces various toxic Aβ species in the brain
Source: J Nanobiotechnology. 2023 Jan 31;21:36. doi: 10.1186/s12951-023-01772-y (PMC9888736; doi:10.1186/s12951-023-01772-y)
Supplement: Supplementary file 3 — Additional file 3. Tables of PMs and data availability of the study. Table S1. DLS Analysis of Fabs Encapsulated in the PMs. Table S2. Quantitative values of the results. [file 12951_2023_1772_MOESM3_ESM.pdf]

Additional file 3: Table S1. DLS Analysis of Fabs Encapsulated in the PMs

	Size (mean ± SEM[a])[b] (nm)	Polydispersity index	ζ Potential (mean ±SEM[a])[b] (mV)
6H4 Fab PM	52.0 ± 7.55	0.169 ± 0.007	-2.03 ± 0.50
3D6 Fab PM	50.2 ± 8.89	0.152 ± 0.041	-1.95 ± 0.46

[a] Standard error of the mean (SEM) (n=3).
[b] Each value was calculated using the cumulant method.

DLS, dynamic light scattering; Fabs, antibody fragments; PMs, polymeric nanomicelles

Additional file 3: Table S2. Quantitative values of the results

Classified group	PBS	6H4 Fab PM	3D6 Fab PM	6H4 Fab	WT
**Quantitative evaluation of Aβ species**					
Aβ40 (pmol/mg protein)					
Number	7	9	8	5	5
Mean	2.8	1.5	1.7	2.5	0.1
Standard error	0.4	0.2	0.2	0.8	0.0
p-value (vs. PBS)	-	0.04	0.10	0.90	-
Aβ42 (pmol/mg protein)					
Number	7	9	8	5	5
Mean	9.9	5.2	4.8	7.5	0.0
Standard error	0.8	0.5	0.5	2.2	0.0
p-value (vs. PBS)	-	0.006	0.003	0.394	-
AβO (pmol/mg protein)					
Number	4	4	4	4	5
Mean	82.8	28.8	45.5	40.0	16.4
Standard error	21.8	7.3	1.6	5.6	2.8
p-value (vs. PBS)	-	0.03	0.17	0.10	-
Toxic conformer (pg/mg protein)					
Number	6	6	6	5	4
Mean	12.2	3.4	5.6	7.5	0.1
Standard error	1.6	0.5	0.7	2.1	0.1
p-value (vs. PBS)	-	0.001	0.009	0.110	-
Insoluble N3pE Aβ40 (pg/mg protein)					
Number	5	6	6	5	5
Mean	0.99	0.03	0.46	0.91	0.00
Standard error	0.30	0.01	0.13	0.48	0.00
p-value (vs. PBS)	-	0.04	0.31	0.99	-
Insoluble N3pE Aβ42 (pg/mg protein)					
Number	5	4	6	5	5

Mean	10.3	6.4	8.4	8.3	0.1
Standard error	1.1	0.1	0.5	0.9	0.0
p-value (vs. PBS)	-	0.01	0.24	0.22	-
Soluble N3pE Aβ42 (pg/mg protein)					
Number	5	5	4	5	4
Mean	0.65	0.28	0.44	0.74	0.06
Standard error	0.11	0.07	0.09	0.11	0.04
p-value (vs. PBS)	-	0.04	0.35	0.81	-
82E1 positive plaque diameter (µm)					
Number	3	4	3	3	3
Mean	46.5	31.2	40.4	37.8	8.9
Standard error	2.0	2.0	3.6	2.3	4.8
p-value (vs. PBS)	-	0.001	0.346	0.161	-
82E1 positive area (%ROI)					
Number	3	3	3	3	3
Mean	3.9	1.3	3.9	3.5	0.2
Standard error	0.2	0.2	0.5	0.8	0.0
p-value (vs. PBS)	-	0.01	1.00	0.85	-
6H4 positive area (%ROI)					
Number	3	4	3	3	3
Mean	4567	2165	4473	6386	517
Standard error	320	281	177	1152	275
p-value (vs. PBS)	-	0.04	1.00	0.14	-
Thioflavin S positive area (%ROI)					
Number	3	4	3	3	3
Mean	2036	862	1128	967	189
Standard error	470	276	106	155	142
p-value (vs. PBS)	-	0.04	0.14	0.08	-
N3pE Aβ positive area (%ROI)					
Number	4	3	3	3	3

Mean	1.67	0.38	1.05	1.03	0.00
Standard error	0.34	0.09	0.29	0.12	0.00
p-value (vs. PBS)	-	0.02	0.27	0.25	-
N3pE Aβ diameter (μm)					
Number	4	3	3	3	3
Mean	63.9	26.4	50.4	56.0	0.0
Standard error	4.2	2.6	6.3	4.6	0.0
p-value (vs. PBS)	-	0.001	0.151	0.509	-
Soluble Aβ40 (pmol/mg protein)					
Number	5	7	6	5	5
Mean	0.021	0.021	0.022	0.023	0.000
Standard error	0.005	0.002	0.004	0.002	0.000
p-value (vs. PBS)	-	>0.99	0.98	0.97	-
Soluble Aβ42 (pmol/mg protein)					
Number	5	9	8	5	5
Mean	0.036	0.031	0.037	0.040	0.000
Standard error	0.008	0.005	0.003	0.007	0.000
p-value (vs. PBS)	-	0.81	1.00	0.96	-
**Behavioral tests**					
Time (sec) of the hidden platform test					
Day 1					
Number	5	8	8	5	3
Mean	170.5	87.4	157.3	147.6	42.7
Standard error	9.5	27.7	15.0	32.4	10.5
p-value (vs. PBS)	-	0.07	0.79	0.83	-
Day 2					
Mean	153.8	44.4	77.9	62.2	29.7
Standard error	16.3	13.8	23.6	32.9	3.3
p-value (vs. PBS)	-	0.002	0.054	0.105	-
Day 3					
Mean	162.5	71.0	77.0	56.2	26.3

Standard error	13.7	24.7	32.8	31.6	10.8
p-value (vs. PBS)	-	0.03	0.11	0.06	-
Cumulative time on platform (%) in the probe test					
Start					
Number	4	6	6	4	3
Mean	21.4	22.0	23.0	22.8	38.1
Standard error	3.8	3.8	8.0	10.1	4.0
p-value (vs. PBS)	-	>0.99	>0.99	>0.99	-
Side 1					
Mean	25.5	7.1	24.1	10.8	12.9
Standard error	8.6	2.5	10.0	5.6	5.5
p-value (vs. PBS)	-	0.18	>0.99	0.73	-
Side 2					
Mean	18.9	14.1	23.3	20.1	13.4
Standard error	2.9	3.8	7.2	5.4	3.1
p-value (vs. PBS)	-	>0.99	>0.99	>0.99	-
Plat form					
Mean	24.8	57.1	24.1	43.9	45.0
Standard error	9.8	5.2	4.6	17.5	1.5
p-value (vs. PBS)	-	0.04	>0.99	0.85	-
Spontaneous alteration rate in Y-maze test (%)					
Number	7	8	8	5	5
Mean	43.4	47.5	43.9	44.8	43.6
Standard error	5.6	4.1	4.1	5.1	5.4
p-value (vs. PBS)	-	>0.99	>0.99	>0.99	-

AβO, amyloid β oligomer; Fab, antibody fragment; PBS, phosphate-buffered saline; PM, polymeric nanomicelle; ROI, region of interest; WT, wild type
